# Biomechanical Properties of Mouse Carotid Arteries With Diet-Induced Metabolic Syndrome and Aging

**DOI:** 10.3389/fbioe.2022.862996

**Published:** 2022-03-22

**Authors:** Anastasia Gkousioudi, Xunjie Yu, Jacopo Ferruzzi, Juncheng Qian, Richard D. Wainford, Francesca Seta, Yanhang Zhang

**Affiliations:** ^1^ Department of Mechanical Engineering, Boston University, Boston, MA, United States; ^2^ Department of Bioengineering, The University of Texas at Dallas, Richardson, TX, United States; ^3^ Department of Pharmacology and Experimental Therapeutics, The Whitaker Cardiovascular Institute, Boston University School of Medicine, Boston, MA, United States; ^4^ Vascular Biology Section, The Whitaker Cardiovascular Institute, Boston University School of Medicine, Boston, MA, United States; ^5^ Division of Materials Science and Engineering, Boston University, Boston, MA, United States; ^6^ Department of Biomedical Engineering, Boston University, Boston, MA, United States

**Keywords:** metabolic syndrome, aging, elastic energy storage, material stiffness, biaxial inflation-extension

## Abstract

Metabolic syndrome increases the risk of cardiovascular diseases. Arteries gradually stiffen with aging; however, it can be worsened by the presence of conditions associated with metabolic syndrome. In this study, we investigated the combined effects of diet-induced metabolic syndrome and aging on the biomechanical properties of mouse common carotid arteries (CCA). Male mice at 2 months of age were fed a normal or a high fat and high sucrose (HFHS) diet for 2 (young group), 8 (adult group) and 18–20 (old group) months. CCAs were excised and subjected to *in vitro* biaxial inflation-extension tests and the Cauchy stress-stretch relationships were determined in both the circumferential and longitudinal directions. The elastic energy storage of CCAs was obtained using a four-fiber family constitutive model, while the material stiffness in the circumferential and longitudinal directions was computed. Our study showed that aging is a dominant factor affecting arterial remodeling in the adult and old mice, to a similar extent, with stiffening manifested with a significantly reduced capability of energy storage by ∼50% (*p* < 0.05) and decreases in material stiffness and stress (*p* < 0.05), regardless of diet. On the other hand, high fat high sucrose diet resulted in an accelerated arterial remodeling in the young group at pre-diabetic stage by affecting the circumferential material stiffness and stress (*p* < 0.05), which was eventually overshadowed by aging progression. These findings have important implications on the effects of metabolic syndrome on elastic arteries in the younger populations.

## Introduction

Metabolic syndrome is a major risk factor for cardiovascular diseases (CVD), such as coronary heart disease and heart attack, and is associated with hypertension and diabetes (Grundy, 2004; Weisbrod et al., 2013; Hall et al., 2015). Metabolic syndrome is strongly linked with obesity and specifically the accumulation of intra-abdominal fat (Han and Lean, 2016; Engin, 2017). According to the World Health Organization, the prevalence of obesity tripled worldwide since 1975 with more than 650 million people being identified as obese. The increasing prevalence of obesity in adults and children has rendered it an epidemic, which can have severe consequences for the cardiovascular system (Poirier et al., 2006; Aroor et al., 2018). Obesity promotes hypertension through the activation of the sympathetic nervous system as well as secretion of inflammatory cytokines from the adipose tissue and hormonal signaling dysregulation, manifested mainly with hyperglycemia and insulin resistance (Grundy, 2004; Rahmouni et al., 2005; Ritchie and Connell, 2007; Kotsis et al., 2010; Weisbrod et al., 2013). Each of the aforementioned mechanisms are major independent risk factors for CVD and their co-occurrence is commonly referred to as metabolic syndrome (Grundy, 2004; Ritchie and Connell, 2007).

Large arteries stiffen with aging and obesity (Gaballa et al., 1998; Safar et al., 2006; Aroor et al., 2018). Arterial stiffness is an independent factor for cardiovascular complications (Amar et al., 2001; Mattace-Raso et al., 2006; Weisbrod et al., 2013; Chirinos et al., 2019; Boutouyrie et al., 2021). The amount and structural organization of the extracellular matrix (ECM) components, collagen and elastic fibers, largely define the passive mechanical properties of the arterial wall, including its stiffness (Wagenseil and Mecham, 2009; Chow et al., 2013; Chow et al., 2014; Zeinali-Davarani et al., 2015; Wang et al., 2016; Cocciolone et al., 2018; Yu et al., 2018). Arterial stiffening in aging is often seen as a result of increased collagen deposition and cross-linking, as well as deterioration of elastic fibers (Poirier et al., 2006; Bonnans et al., 2014; Kohn et al., 2015). Arterial stiffening in aging is accelerated in the presence of other cardiovascular risk factors, such as diabetes and obesity (Lee and Oh, 2010), although the specific pathophysiological mechanisms of obesity-induced arterial stiffness are not fully elucidated. Development of advanced glycation end-products (AGEs), as well as low-grade inflammation and impaired endothelial function are well-known contributors to increased arterial stiffness in metabolic syndrome (Aroor et al., 2018). Regardless of the pathogenesis, arterial stiffness is associated with increased systolic and pulse pressures and often results in the development of hypertension (Weisbrod et al., 2013; Humphrey et al., 2016).

Numerous animal models have been used to study vascular remodeling in aging and the development of various cardiovascular diseases. In these studies, the stiffness of the arterial wall is measured *in vivo* as pulse wave velocity, compliance and distensibility (Stehouwer et al., 2008; Spronck and Humphrey, 2019), or *in vitro* in excised vessels, with some of the most commonly used measures being material and structural stiffness (Ferruzzi et al., 2013). Material stiffness is an intrinsic property that correlates mechanobiological responses with stress and strain fields (Ferruzzi et al., 2013). The combination of material stiffness and wall dimensions, i.e., the structural stiffness, were suggested to impact the hemodynamic wave propagation (Baek et al., 2007; Ferruzzi et al., 2013; Spronck and Humphrey, 2019). Given that the main function of large elastic arteries is to facilitate blood flow, elastic stored energy was shown to be a more effective parameter in measuring arterial stiffening, rather than material or structural stiffness (Ferruzzi et al., 2015).

To understand how the coexistence of cardiovascular risk factors such as obesity and diabetes affect the mechanical behavior of large elastic arteries, here we studied the effects of high fat high sucrose (HFHS) diet and aging on the biomechanics of central elastic arteries of mice. Common carotid arteries (CCA) were isolated from mice fed a normal or HFHS diet for 2, 8 and 18–20 months and subjected to biaxial inflation-extension tests, from which the stress-stretch relationships in circumferential and longitudinal directions were determined. Elastic energy storage was then calculated as a function of the applied transmural pressure by fitting a four-fiber family model to the biaxial stresses-stretch responses, while the material stiffness was also computed of each group.

## Materials and Methods

### Animal Model of Metabolic Syndrome

All animal procedures were approved by the Boston University Institutional Animal Care and Use Committee (IACUC). Male C57Bl/6J mice (stock number 00664) were purchased from the Jackson Laboratory (Bar Harbor, ME, United States) at 7 weeks of age. After 1 week of acclimatation, mice were fed a control normal diet (ND: 4.5% fat, 0% sucrose, catalog number D09071702, Research Diets, New Brunswick, NJ, United States) or high fat, high sucrose diet (HFHS: 35.5% fat, 16.4% sucrose) ad libitum (catalog numbers D09071703, Research Diets, New Brunswick, NJ, United States) (Weisbrod et al., 2013). The normal diet is formulated to match the micronutrients contained in the HFHS diet, except for fat and sucrose, and to provide comparable kcal/g as the HFHS diet. We characterized this model of diet-induced metabolic syndrome (Weisbrod et al., 2013; Fry et al., 2016), which develops hyperinsulinemia, glucose-intolerance, arterial stiffness and hypertension within 8 months, closely mimicking the human metabolic syndrome. Mice were fed ND or HFHS for 2, 8 and 18–20 months as per the following experimental groups (*n* = 5 unless otherwise noted): 2 months/ND, 2 months/HFHS, 8 months/ND, 8 months/HFHS (*n* = 4), 20 months/ND (*n* = 7), 20 months/HFHS (*n* = 7). The mouse body weights were recorded weekly. Throughout this study, 2 months mice are denoted as “young” mice, 8 months mice are denoted as “adult” mice and 20 months are denoted as “old”, regardless of diet. To summarize, the body weights for the ND groups were 29.68 ± 1.19 g, 40 ± 2.51 g, and 43.68 ± 2.84 g for the young, adult and old groups, respectively; and for the HFHS groups were 38 ± 3.66 g, 50 ± 3.03 g, and 57.63 ± 4.3 g for the young, adult and old groups, respectively. Blood pressure was measured using radio telemetry, which consists of a pressure-sensing gel-filled catheter that was inserted in the aortic arch via catheterization of the left carotid artery and a wireless transmitter (Weisbrod et al., 2013). A continuous 20-s recording of the blood pressure wave was acquired every hour. Daily recordings for each mouse were averaged before averaging different experimental groups. The mean arterial pressure (MAP) for the ND groups were 104 mmHg, 111 and 120 mmHg for the young, adult and old groups, respectively; and for the HFHS groups were 108, 115 and 125 mmHg for the young, adult and old groups, respectively.

### Biaxial Inflation-Extension Test

Carotid arteries were excised from each mouse and carefully cleaned from surrounding connective tissue and fat. Segments of 5 mm were cut from each artery and stored in phosphate buffered saline (PBS). All arteries were mechanically tested within 12 h of dissection. Biaxial inflation-extension test was performed using a pressure myograph (110P DMT, Danish Myo Technology, Denmark). Each artery was carefully cannulated and secured in the chamber with 7–0 suture, while being immersed in a PBS bath at room temperature throughout the mechanical testing. Arteries were preconditioned by undergoing an axial stretch of 1.8 and pressurization from 0 to 140 mmHg three times, to ensure consistent behavior during mechanical testing (Ferruzzi et al., 2013). After preconditioning, the *in vivo* stretch ratio of each artery was estimated by inflating the artery at three different pressure values (50, 90 and 120 mmHg) and axially stretching it from $\lambda_{z}$ = 1 to $\lambda_{z}$ = 2 with 0.1 increments at each pressure value, resulting in three axial stretch-axial force curves. The *in vivo* stretch ratio was identified as the intersection point of these three curves (Weizsäcker et al., 1983). Arteries were then subjected to biaxial inflation-extension tests at the *in vivo* axial stretch ratio, $\lambda_{z}^{iv}$ , as well as 5% above and below the *in vivo* stretch ratio, $\lambda_{z}^{+}$ and $\lambda_{z}^{-}$ , respectively. The loading protocol consisted of a stepwise inflation and deflation of the artery from 0 to 140 mmHg with 10 mmHg increments and a loading rate of 5 mmHg/sec. The axial force, outer diameter and applied pressure were recorded during the testing. After mechanical testing, the unloaded arterial dimensions were measured by imaging arterial rings of ∼0.5 mm in length. The images were then imported into FIJI (http:/Fiji.sc/Fiji, Ashburn, VA). The outer and inner circumference were manually traced, and the corresponding diameters were calculated.

Under thin wall assumption, Cauchy stresses in the circumferential and longitudinal directions can be calculated as (Humphrey and Na, 2002):

$$\sigma_{\theta }=\frac{Pr_{i}}{h}\;\mathrm{and}\;\sigma_{z}=\frac{f+P\pi r_{i}^{2}}{\pi h\left(2r_{i}+h\right)}$$ (1)

where P is the transmural pressure, f is the axial force, $r_{i}$ is the deformed inner radius and h is the deformed thickness. Subscripts θ and z refer to the circumferential and longitudinal direction of the arterial wall, respectively. With incompressibility assumption, the deformed inner radius can be calculated as (Ferruzzi et al., 2013):

$$r_{i}=\frac{1}{2}\sqrt{d_{o}^{2}-\frac{D_{o}^{2}-D_{i}^{2}}{\lambda_{z}}}$$ (2)

where $d_{o}$ is the deformed outer diameter, $\lambda_{z}$ the axial stretch ratio and $D_{o}$ and $D_{i}$ are the undeformed outer and inner diameter, respectively.

### Constitutive Modeling

A structurally motivated four-fiber family constitutive model was used to describe the passive mechanical behavior of the arteries. In this model the arterial wall is considered a composite of the anisotropic collagen fibers and the isotropic arterial matrix, i.e., elastic fibers, cells and ground substance. The strain energy function (SEF) has the following form (Ferruzzi et al., 2013):

$$W\left(C,a^{i}\right)=\frac{c}{2}\left(I_{C}-3\right)+\overset{4}{\underset{i=1}{\sum }}\frac{c_{1}^{i}}{4c_{2}^{i}}\left\{exp\left[c_{2}^{i}{\left(I_{4}^{i}-1\right)}^{2}\right]-1\right\}$$ (3)

where c is a material parameter for the isotropic matrix, $c_{1,2}^{i}$ are material parameters for the collagen fibers, and $I_{C}=trC$ is the first invariant of the Cauchy-Green deformation tensor $C=F^{T}F$ with $F=diag\left(\frac{\partial r}{\partial R},\frac{r}{R},\lambda_{z}\right)$ being the deformation gradient from the undeformed (R,Θ,Z) to deformed configuration (r,θ,z) . $I_{4}^{i}=a^{i}\cdot Ca^{i}$ represents the invariant that is associated with the $i^{th}$ fiber family, and $a^{i}$ is the unit vector that denotes the orientation of the $i^{th}$ fiber family. The collagen fibers are assumed to be oriented in the axial ( $\varphi^{1}$ = 0^o^), circumferential ( $\varphi^{2}$ = 90^o^) and diagonal ( $\varphi^{3}=-\varphi^{4}=\varphi$ ) direction. The Cauchy stress can be then calculated as:

$$t=-pI+2F\frac{\partial \text{W}}{\partial C}F^{T}$$ (4)

where p is a Lagrange multiplier, I is the identity matrix and W is the SEF in Eq. 3. The Lagrange multiplier, p , was estimated based on the plane stress assumption, in which the radial stress is considered to be zero.

### Determination of Model Parameters

The longitudinal and circumferential Cauchy stress-stretch responses from the model derived from Eq. 4 were fitted to the experimental data for all three stretch ratios ( $\lambda_{z}^{iv},\;\lambda_{z}^{+},\;\lambda_{z}^{-}$ ). All calculations were performed in the unloading phase which is assumed to include only the stored strain energy that the arteries use to elastically recoil during diastole. In total *n* = 6 curves were used in the fitting process for every artery, one for every stretch ratio and direction, in order to estimate the eight model parameters, i.e., $c,\;c_{1}^{1},c_{2}^{1},\;c_{1}^{2},\;c_{2}^{2},\;c_{1}^{3,4},c_{2}^{3,4},\phi$ , in MATLAB using the function *fminsearch* and the Nelder-Mead algorithm by minimizing the following objective function:

$$e=\overset{N}{\underset{i=1}{\sum }}\left[{\left(\sigma_{\theta \theta }^{m}-\sigma_{\theta \theta }^{e}\right)}_{i}^{2}+{\left(\sigma_{zz}^{m}-\sigma_{zz}^{e}\right)}_{i}^{2}\right]$$ (5)

where N is the number of the total data points of the stress-stretch curves, and superscripts m and e denote model and experiment, respectively. Once the best-fitted parameter values were determined, the SEF was calculated using Eq. 3.

### Calculation of Material Stiffness

Material stiffness was obtained using the theory of small deformations superimposed on large at MAP for each group as (Baek et al., 2007; Ferruzzi et al., 2013):

$${\mathbb{C}}_{ijkl}=2\delta_{ik}F_{lA}^{o}\frac{\partial W}{\partial C_{AB}}F_{jB}^{o}+2\delta_{jk}F_{iA}^{o}\frac{\partial W}{\partial C_{AB}}F_{lB}^{o}+4F_{iA}^{o}F_{jB}^{o}F_{kP}^{o}F_{lQ}^{o}{\frac{\partial^{2}W}{\partial C_{AB}\partial C_{PQ}}|}_{C^{o}}$$ (6)

where $F^{o}$ is the deformation gradient and $C^{o}$ is the associated Cauchy-Green deformation tensor.

### Statistical Analysis

All data are presented as mean ± standard error of the mean (SEM). A two-way analysis of variance (ANOVA) was used to access statistical differences due to the independent factors of age and diet, as well as their interaction. Post hoc analyses were performed using Tukey’s test to determine the effect of age within diet (i.e., young vs. adult, adult vs. old and young vs. old) and the effect of diet within each age group (i.e., ND vs. HFHS). A value of *p* < 0.05 was considered significant. All statistical analyses were performed using IBM SPSS (Version 26.0).

## Results

The arterial dimensions of CCA in the unloaded configuration for the diet (ND and HFHS) and age (young, adult and old) groups were summarized in Table 1. Overall, there is a significant change in the inner diameter and arterial wall thickness from the young to the adult and old ND arteries (*p* < 0.05). For the HFHS groups, the changes in the outer and inner diameters from the young to the adult group are not significant, however these changes cumulatively resulted in a significant increase in the thickness of the adult arteries (*p* < 0.05). There are no significant changes in arterial dimensions from the adult to old groups, and between the ND and HFHS arterial dimensions within each age group.

**TABLE 1:** The arterial dimensions of CCA (mean ± SEM) in the unloaded configuration for the diet (ND and HFHS) and age (young, adult and old) groups (*p < 0.05 represents comparisons with the young group).

	ND	HFHS
Young		
Outer Diameter (μm)	487.59 ± 5.14	477.85 ± 17.66
Inner Diameter (μm)	322.83 ± 4.13	309.4 ± 17.87
Thickness (μm)	82.38 ± 4.45	84.22 ± 4.46
Adult		
Outer Diameter (μm)	519.42 ± 3.83	511.36 ± 10.53
Inner Diameter (μm)	295.14 ± 6.46^*^	284.97 ± 10.01
Thickness (μm)	112.14 ± 3.45^*^	113.2 ± 4.02^*^
Old		
Outer Diameter (μm)	491.02 ± 11.13	495.29 ± 15
Inner Diameter (μm)	285.21 ± 6.2^*^	293.07 ± 13.49
Thickness (μm)	102.91 ± 3.28^*^	101.11 ± 3.17^*^

**FIGURE 1:**
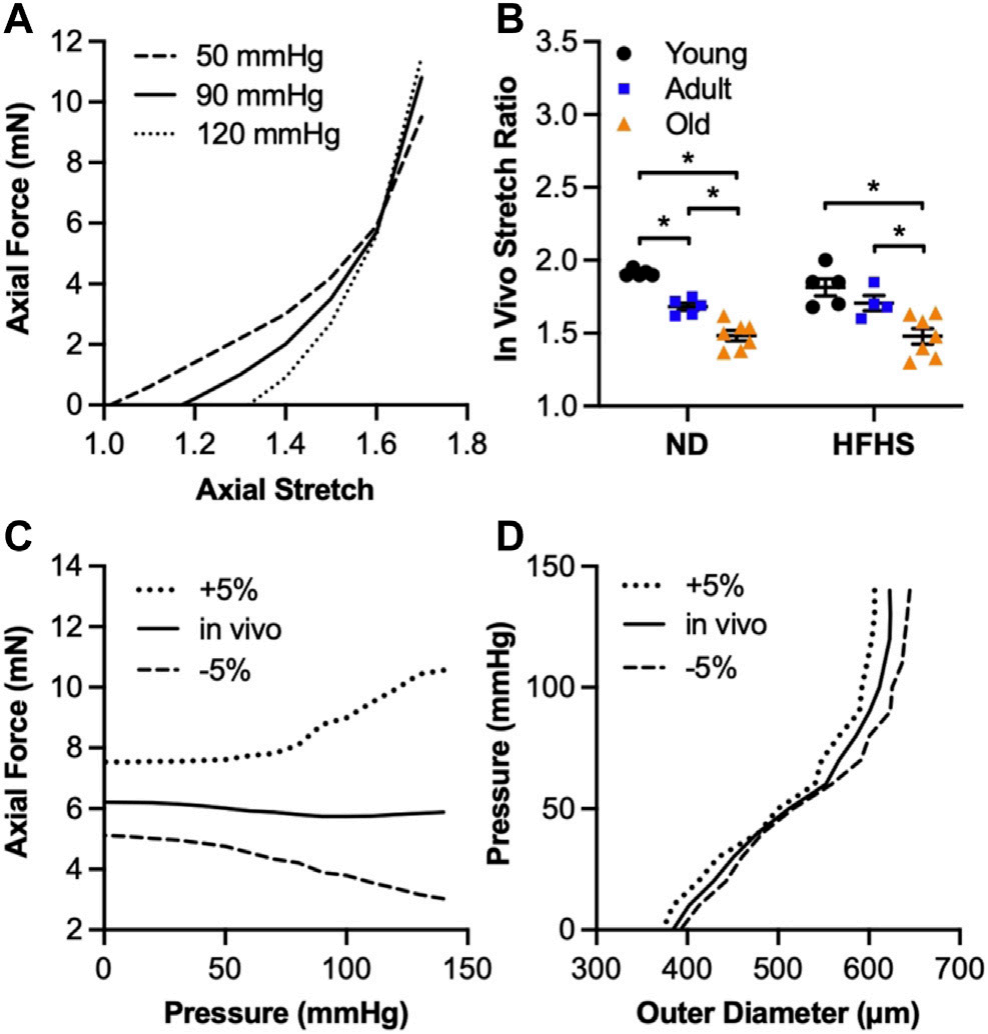
*In vivo* axial stretch ratio and typical arterial response under a pressure-diameter testing. **(A)**: Estimation of the *in vivo* axial stretch ratio as the intersection point of the three axial force-stretch curves at different pressure values (50 mmHg, 90 mmHg, 120 mmHg). **(B)**: Estimated *in vivo* ratio for the age (young, adult and old) and diet (ND and HFHS) groups. **(C,D)**: Typical axial force-pressure and pressure-outer diameter response of arteries at three different axial stretches (*in vivo*, 5% above and below *in vivo*) (*p < 0.05).

Figure 1A shows representative axial force-axial stretch responses of an adult ND mouse carotid artery at 50, 90, and 120 mmHg intraluminal pressures. The intersection point of the three curves was used to estimate the *in vivo* axial stretch ratio. The averaged *in vivo* stretch ratio for each group is shown Figure 1B. Aging resulted in a consistent decrease in the *in vivo* stretch ratio regardless of the diet, from 1.91 ± 0.01 to 1.68 ± 0.03 (*p* < 0.05) and then to 1.48 ± 0.03 (*p* < 0.05) for the young, adult, and old ND arteries, respectively; and from 1.82 ± 0.06 to 1.70 ± 0.05 and then to 1.48 ± 0.05 (*p* < 0.05) for the young, adult, and old HFHS arteries, respectively. There was no significant decrease between the *in vivo* stretch ratios of the ND and HFHS arteries within each age group. The artery was then subjected to pressurization while being stretched at the *in vivo* stretch ratio and 5% stretch above and below the estimated *in vivo* axial stretch ratio, during which the axial force and outer diameter was recorded. Figures 1C,D show the representative responses of axial force and outer diameter as a function of intraluminal pressure for the same artery in Figure 1A. The almost constant axial force vs. increasing pressure (Figure 1C) was used as a confirmation of the estimated *in vivo* stretch ratio, while higher and lower *in vivo* axial stretch ratios resulted in an increasing and decreasing trend in the axial forces, respectively (Ferruzzi et al., 2013). Figure 1D shows a representative outer diameter as a function of pressure for the three different axial stretch ratios. When the artery was stretched axially 5% less than its *in vivo* stretch, it was more inflated under the same intraluminal pressure which resulted in a slightly larger outer diameter. The opposite trend is observed for a 5% greater stretch ratio.

**FIGURE 2:**
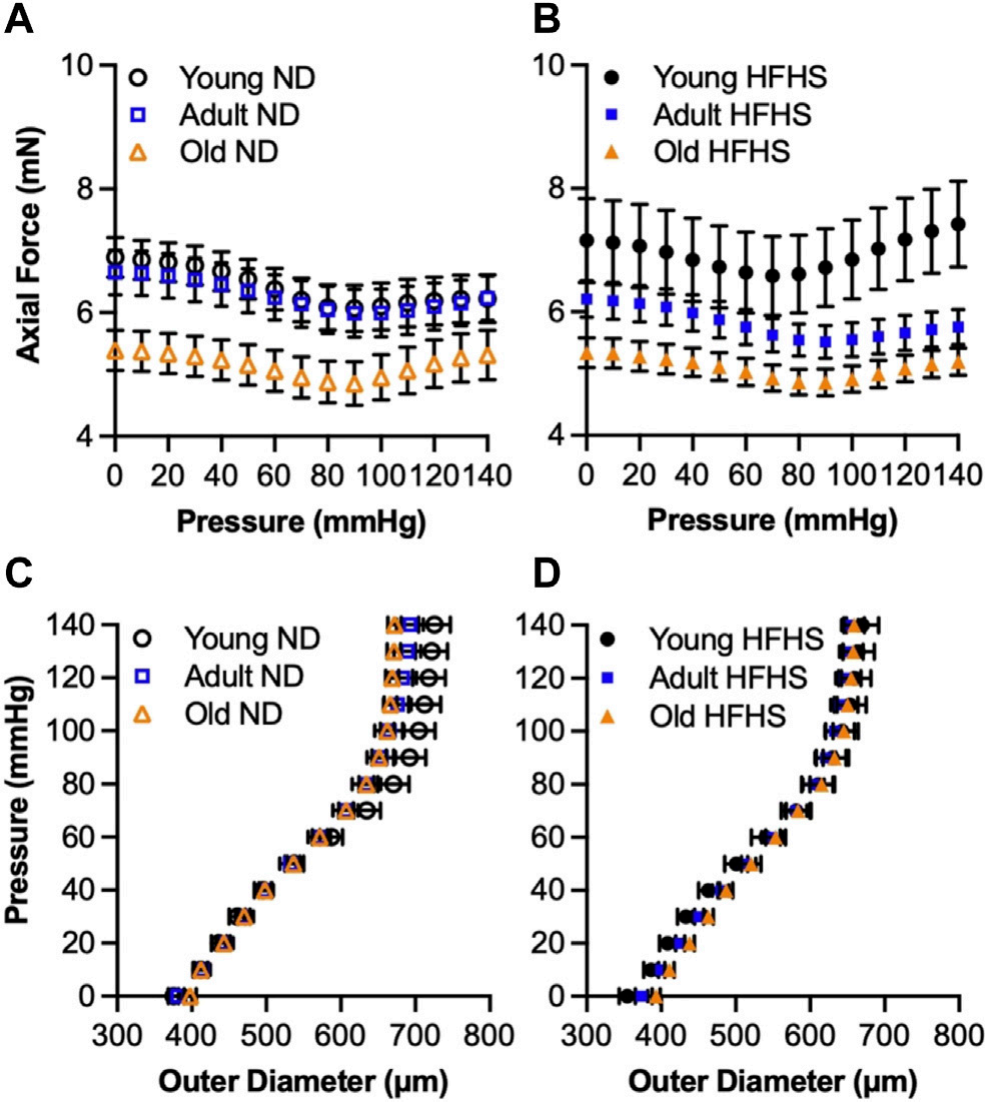
Average (mean ± SEM) axial force-pressure **(A,B)** and pressure-outer diameter **(C,D)** responses at *in vivo* axial stretch ratio for the age (young, adult and old) and diet (ND and HFHS) groups.

During biaxial inflation-extension test, the artery was first axially stretched to the estimated *in vivo* stretch ratio and then subjected to pressurization up to 140 mmHg. Figures 2A,B show the axial force needed to maintain the carotid arteries at their *in vivo* axial stretch throughout the applied pressure range, as a function of diet and age. Overall, there was a decreasing trend in axial force with aging, and this trend was more obvious in the old ND and HFHS groups. The young HFHS arteries responded with a higher axial force than the ND ones, especially when the pressure was higher than 80 mmHg. There was no obvious difference in axial force for the adult and old arteries between the ND and HFHS groups. For the adult group, however, the axial force of the HFHS arteries was consistently slightly lower than the ND arteries. The axial force of the old ND and HFHS arteries was almost indistinguishable. The pressure-diameter curves in Figures 2C,D showed a leftward shift with aging and diet. The ND arteries appeared to be more distensible than HFHS arteries for the young and adult groups, however this trend diminished with aging as the old ND and HFHS responded similarly under pressure.

**FIGURE 3:**
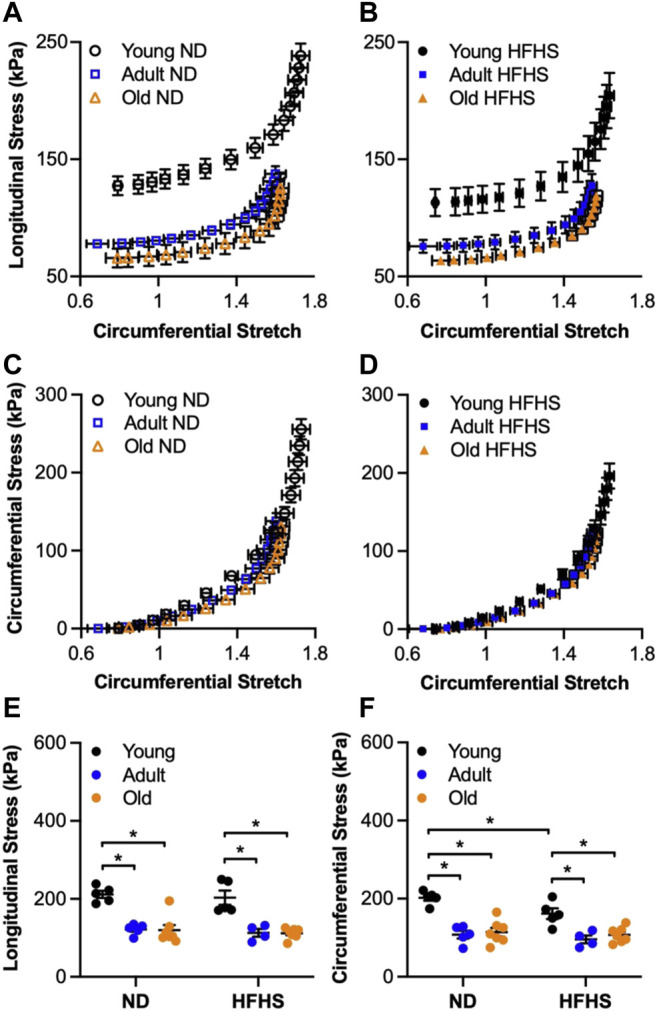
Average (mean ± SEM) experimental longitudinal **(A,B)** and circumferential **(C,D)** Cauchy stress-stretch responses for the age (young, adult and old) and diet (ND and HFHS) groups. Cauchy stress at MAP in longitudinal **(E)** and circumferential **(F)** direction as a function of age (young, adult and old) and diet (ND and HFHS) (*p < 0.05).

The Cauchy stresses vs. circumferential stretch curves for the four experimental groups are shown in Figures 3A–D. Diet had an early effect characterized by a downward and leftward shift of the longitudinal stress for the young HFHS arteries when compared to the young ND arteries (Figures 3A,B). In the circumferential direction, the young HFHS arteries responded to pressurization with lower stresses compared to the ND arteries and a slightly leftward shift for higher circumferential stretches (Figures 3C,D). The Cauchy stresses were also plotted at MAP of each group, in both directions and are shown in Figures 3E,F. Aging resulted in a marked decrease in longitudinal and circumferential stresses for both ND and HFHS arteries. The longitudinal stresses decreased from 206.67 ± 10.96 kPa for young ND to 118.2 ± 5.89 kPa for adult ND arteries (*p* < 0.05), and from 175.8 ± 19 kPa for young HFHS to 110.91 ± 10 kPa for adult HFHS arteries (*p* < 0.05) (Figure 3E). When compared to the young arteries, old arteries showed a significant decrease as well to 108.22 ± 11.46 kPa and 102.6 ± 4.57 kPa for the ND and HFHS, respectively (*p* < 0.05). Similar results were shown in the circumferential stresses (Figure 3F) which decreased from 192.88 ± 11.7 kPa for young ND to 102.08 ± 9.02 kPa for the adult ND arteries (*p* < 0.05) and from 146.03 ± 13.75 kPa to 92.3 ± 8.67 kPa for the HFHS arteries at the same age groups (*p* < 0.05). Similarly to the longitudinal direction, circumferential stress of the old arteries decreased significantly to 100.77 ± 9.08 kPa for the ND and 93.41 ± 5.57 kPa for the HFHS diet group, when compared to the young ones (*p* < 0.05). Interestingly, there was no significant difference in stresses between the adult and the old groups. In terms of the effect of diet within each age group, significant decrease in circumferential stress for the young arteries due to HFHS diet was observed (*p* < 0.05).

**FIGURE 4:**
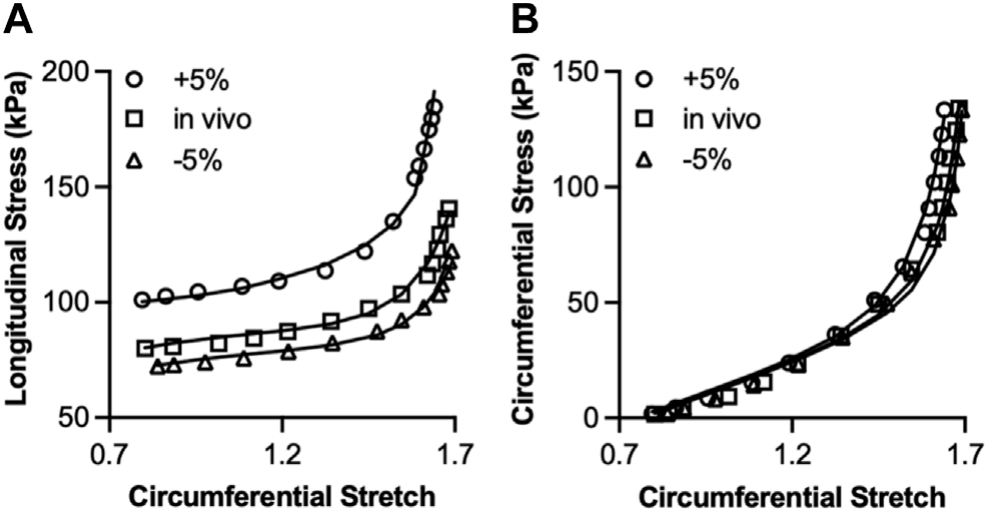
Fitting of a 4-fiber family model to the longitudinal **(A)** and circumferential **(B)** Cauchy stress-stretch curves at three different axial stretches (*in vivo*, 5% above and below *in vivo*). The equations for the longitudinal and circumferential Cauchy stresses were fitted to the experimental data under the three axial stretches simultaneously. Best-fit parameters and coefficient of determination, $R^{2}$ are given in Supplementary Tables S1–S3.

A representative fitting of the stress-stretch responses from the model to the experimental data is shown in Figure 4 for the three different axial stretches. The model parameters were determined by fitting the longitudinal (Figure 4A) and circumferential stress-stretch (Figure 4B) responses at the three longitudinal stretch ratios simultaneously. The model captured well the arterial passive response in both directions for all axial stretches. The best-fit parameters and the coefficient of determination, $R^{2}$ are given in Supplementary Tables S1–S3.

**FIGURE 5:**
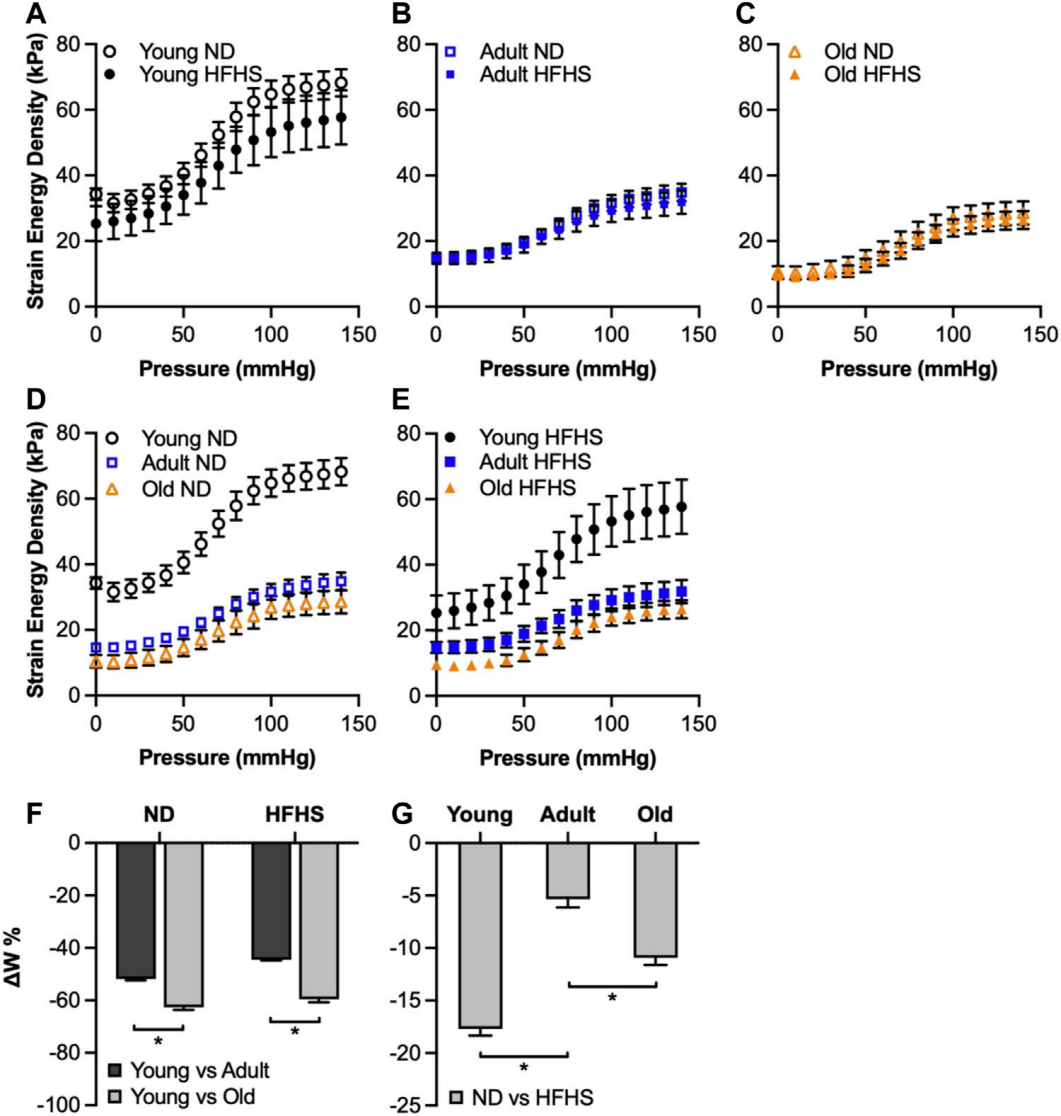
Comparison of theoretical calculated strain energy density (mean ± SEM) as a function of age (young, adult, and old) and diet (ND and HFHS). **(A–E)**: Theoretical calculated strain energy density as a function of the applied pressure. The five sub-figures show the effect of diet **(A–C)** and aging **(D,E)** on strain energy. The bar graphs **(F,G)** show the averaged (mean ± SEM) normalized difference in strain energy (*p < 0.05).

The elastic stored energy was then calculated using Eq. 3 as a function of the applied transmural pressure. Figures 5A–E show the elastic stored energy density with aging and diet effects. The elastic energy had an almost two-fold increase as pressure increased from 0 to 140 mmHg for all groups, following an S-shaped curve, with a plateau that was reached at higher pressures of about 110 mmHg. The elastic stored energy was significantly affected by aging in both ND and HFHS arteries, but not by diet, although a slight decrease in stored energy was observed in the young group between the HFHS and ND arteries (Figures 5A–C). This aging-associated decrease was significant throughout the applied pressure range of the ND and HFHS arteries (*p* < 0.05, Figures 5D,E); however, a significant decrease in energy for the adult HFHS arteries was manifested for pressures greater than 70 mmHg (*p* < 0.05, Figure 5E).

The normalized difference in elastic energy storage, ΔW, was calculated and averaged over the applied pressure range and plotted in Figures 5F,G representing the aging and diet effect, respectively. In Figure 5F, to study the effect of aging, the difference in energy storage between the young - adult and young—old groups in Figures 5D,E was first normalized by the young group and then averaged over the applied pressure range, for the ND and HFHS diet, respectively. The energy storage decreased dramatically in both adult and old groups for both ND and HFHS arteries with significant higher decrease in the old arteries (*p* < 0.05). In Figure 5G, to study the effect of diet, the averaged strain energy difference between the ND and HFHS arteries in Figures 5A–C was normalized by the corresponding ND groups, and then averaged over the applied pressure range. Diet affected the total energy storage early on in the age groups, with the young HFHS storing ∼17% less energy than the ND arteries in the same age group, while old HFHS stored ∼10% less energy than the ND ones. Adult arteries were also affected with the HFHS arteries storing also ∼5% less energy than the ND ones.

**FIGURE 6:**
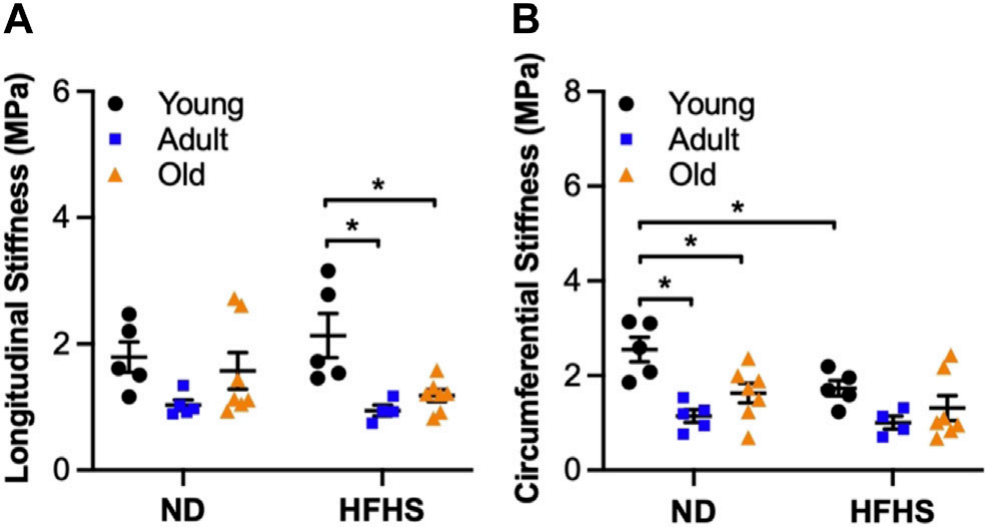
Average (mean ± SEM) material stiffness at MAP in longitudinal **(A)** and circumferential **(B)** direction as a function of age (young, adult, and old) and diet (ND and HFHS) (*p < 0.05).

The material stiffness was also calculated at MAP for each group using Eq. 6 and is shown in Figures 6A,B, for the longitudinal and circumferential direction, respectively. Aging resulted in a significant decrease in material stiffness in the circumferential direction for the ND groups. Specifically, the circumferential stiffness for the ND arteries decreased from 2.83 ± 0.3 MPa for the young to 1.12 ± 0.13 MPa for the adult (*p* < 0.05) and 1.43 ± 0.17 MPa for the old (*p* < 0.05) when compared with the young group. In the HFHS arteries, similar trend was observed but the change was not significant. The circumferential stiffness decreased from 1.83 ± 0.18 MPa for the young to 0.96 ± 0.12 MPa for the adult and 1.04 ± 0.2 MPa for the old when compared with the young group. Diet had an early impact on circumferential material stiffness, which was shown by a significant decrease between the ND and HFHS groups for the young group (*p* < 0.05, Figure 6B). The longitudinal direction showed a similar trend with the material stiffness decreased in ND groups from 1.93 ± 0.28 MPa for the young to 1.02 ± 0.08 MPa for the adult ND arteries, while in HFHS groups aging resulted in a significant decrease from 2.23 ± 0.38 MPa for young to 0.91 ± 0.08 MPa for adult (*p* < 0.05) and 1.42 ± 0.24 MPa for old arteries (*p* < 0.05). Diet had no effect on longitudinal material stiffness in all age groups (Figure 6A).

## Discussion

In this study, changes in arterial wall biomechanics were studied using a mouse model of diet-induced metabolic syndrome, which became pre-diabetic after 2 months on the HFHS diet with increased body weight, glucose intolerance and insulin resistance as reported in an earlier study (Weisbrod et al., 2013). After 6 months on the HFHS diet, these mice further developed hypertension, closely mimicking the human metabolic syndrome (Weisbrod et al., 2013). Additionally, aged mice older than 20 months old have been used extensively as a model of human aging with hallmarks of vascular aging observed (Ferruzzi et al., 2018; Gioscia-Ryan et al., 2020; Hawes et al., 2020). Our results suggest that aging dominated the changes of the biomechanical behavior of mice carotid arteries (CCAs) by significantly affecting arterial wall stresses and material stiffness and compromising elastic energy storage. Interestingly, diet-induced arterial remodeling was more obvious in the young group indicating that the adverse effects of HFHS diet on the CCAs occurred early in the pre-diabetic stage but was lost with progressing age.

It is well established that aging leads to structural changes in the arterial wall with increases in thickness due to an abundant deposition of ECM components in media and adventitia, wall thickening, and proliferation of vascular smooth muscle cells (Gaballa et al., 1998; Jani and Rajkumar, 2006; Greenwald, 2007; Xu et al., 2017). Several studies quantified the content of the various ECM constituents by performing tissue staining and histological analysis in order to study the effect of aging on the morphological characteristics of mouse arteries (Gaballa et al., 1998; Ferruzzi et al., 2018). These studies, using carotid arteries from young and aged mice, found a significant increase in collagen content and thickening of the arterial wall as a result of aging. In our data, the alterations in the unloaded inner and outer diameter (Table 1) resulted in a significant thickening of the adult carotid arteries, whereas old arteries showed a more modest increase in thickness compared to young ones. However, no difference was observed in arterial dimensions due to the HFHS diet when compared with ND arteries in all age groups (Table 1). A possible explanation for this might be associated with the temporal development of diet-induced arterial remodeling. Billaud et al. (2012) examined the structural and functional properties (i.e. compliance) of mice arteries at earlier stages of obesity. In their study, structural changes in carotid and thoracodorsal arteries were altered rapidly after the 6-week high-fat diet with an elevated content of collagen fibers and glycoproteins compared to their normal control diet. However, compliance of the carotid arteries remained unchanged and, thus the authors suggested that carotid arteries were subjected to structural but no functional changes after a 6-week diet. Altogether, these findings indicate that structural changes due to a high-fat diet could happen as early on as 6 weeks of diet which might reach a plateau as aging progresses, explaining the lack of differences between the diet groups in our study.

Biaxial stress-stretch relationship of the arterial wall was also noticeably affected by aging, while showing modest changes due to the HFHS diet when compared to ND arteries in the same age group (Figure 3). The downward shift in biaxial stresses as a function of aging is in agreement with the results reported by Ferruzzi et al. (2018) for aged mice (25 months old) on a mixed C57BL/6 × 129/svEv genetic background. It has been suggested that cells in the arterial wall alter the ECM protein production and consequently the arterial dimensions, as a response to sustained changes in blood pressure and flow, in order to restore the wall stress and to maintain homeostasis (Greenwald, 2007; Humphrey, 2008; Humphrey et al., 2016). Ferruzzi et al. (2018) attributed the increase in arterial thickness without a change in blood pressure to compromised mechanical homeostasis due to aging. The animal model used in our study develops hypertension after 6 months on the HFHS diet (Weisbrod et al., 2013), which coincides with the thickening and decrease in the lumen diameter of the adult arteries, and consequently the decrease in circumferential stresses. The fact that the old group displayed similar stress levels as the adult group indicates that altered mechanical homeostasis due to aging already occurred in the adult group. Furthermore, our study showed that changes on *in vitro* biaxial stresses due to HFHS diet reached a plateau at early stages of metabolic syndrome and the resulting changes are overshadowed by the more prevailing ones caused by aging.

The mechanical functionality of the arteries was compromised with aging and diet, as manifested with a significant decrease in strain energy storage (Figure 5). During systole, elastic arteries stretch and store strain energy, which is later used during diastole for elastic recoil thereby propelling the blood flow in the downstream circulation (Greenwald, 2007). Our results revealed a significantly reduced capability of the aged carotids to store strain energy (Figures 5D,E). In a study by Ferruzzi et al. (2015), the less stored energy during loading was associated with less distensible arteries and it was interpreted as increased arterial stiffness despite the fact that the slopes of the stress-strain curves, i.e. a metric for the material stiffness, were decreased or not significantly different in mice with severe elastopathy. Indeed, our results showed a significantly decreased material stiffness with aging for both diet groups at MAP (Figure 6). Previously studies suggested that the reduced elastic energy storage during cyclic loading may be due to the viscous arterial ECM components, such as collagen fibers, SMCs and proteoglycans, which are associated with energy dissipation (Ferruzzi et al., 2015; Kim et al., 2017). Therefore, the aging-associated increase in these viscous components in the arterial wall could constitute a potential structural factor responsible for the decreased stored energy and consequently, stiffening of the arteries. Even though there is limited research on how acquired cardiovascular risk factor such as obesity and/or diabetes could affect the remodeling of elastic arteries, there is evidence of remodeling of elastin in adipose tissue of obese individuals and becoming more viscous in the settings of high-glucose levels (Spencer et al., 2011; Wang et al., 2015; Cocciolone et al., 2018). Considering that elastic fibers are long-lived proteins responsible for the elasticity of arteries, the lower energy storage observed in the HFHS arteries could suggest an elastin remodeling (Figures 5A–C), especially in the young group.

### Limitations

The *in vitro* biomechanical testing was performed in a PBS solution bath at room temperature, however the passive mechanical property of the arterial wall is known to display very little differences when tested at room and body temperatures (Kang et al., 1995; Humphrey, 2002). The pressurization was at a lower loading rate than what arteries experience *in vivo* (Murtada et al., 2016). Future study on the rate dependency of the arterial wall is warranted. In addition, arteries are subjected to flow-associated loads, thus they experience shear stress, which was not considered in this study. Future studies should take into consideration the active behavior of the arteries, as well as the pulsatile dynamic loading since the arterial stiffness and the hemodynamics are coupled. Finally, elastin is a long-lived protein and the life expectancy of a mouse surpasses elastin’s half-life which is ∼70 years (Shapiro et al., 1991), suggesting that we should be careful when interpreting aging-related results (Spronck et al., 2020). Lastly, generalizability of our data obtained with carotid arteries to other large elastic arteries, such as the aorta, needs to be confirmed due to regional dependency of arterial remodeling.

## Conclusion

Consumption of a diet high in fat and sugar resulted in increase in conditions of metabolic syndrome in a mouse model. Our study provides some important findings regarding arterial remodeling as a function of diet-induced metabolic syndrome and aging. Aging dominated arterial remodeling and affected tissue homeostasis by an inward remodeling of the arterial wall which resulted in a downward shifting of the biaxial stresses and stiffness. However, carotid arteries became stiffer with aging as it was denoted by the decrease in their ability to store elastic energy in the young group, i.e., after 2 months under HFHS diet, suggesting that diet-induced changes in arterial biomechanics of carotid arteries might take place at the early pre-diabetic stage of metabolic syndrome and are being overshadowed as aging progresses. Our study showed that compromised elastic energy storage and arterial biomechanics, such as stress and material stiffness, affected both adult and old groups to a similar extent. Given the prevalence of obesity and the associated development of CVD, the results of our study could shed light into the effects of obesity and diabetes on elastic arteries and their response under physiological loads, especially in the young and mid-aged populations.

## Data Availability

The original contributions presented in the study are included in the article/Supplementary Material, further inquiries can be directed to the corresponding author.
