# Supplementary material for: Biomechanical Properties of Mouse Carotid Arteries With Diet-Induced Metabolic Syndrome and Aging
Source: Front Bioeng Biotechnol. 2022 Mar 22;10:862996. doi: 10.3389/fbioe.2022.862996 (PMC8980683; doi:10.3389/fbioe.2022.862996)
Supplement: Supplementary file 1 [file DataSheet1.PDF]

(Supplemental Material)

Biomechanical Properties of Mouse Carotid Arteries with Diet-Induced Metabolic Syndrome and Aging

Anastasia Gkousioudi[1], Xunjie Yu[1], Jacopo Ferruzzi[2], Juncheng Qian[1], Richard D. Wainford[3], Francesca Seta[4], and Yanhang Zhang[1,5,6*]

[1]Department of Mechanical Engineering, Boston University, Boston, MA

[2]Department of Bioengineering, The University of Texas at Dallas, Richardson, TX

[3]Department of Pharmacology & Experimental Therapeutics and The Whitaker Cardiovascular Institute, Boston University School of Medicine, Boston, MA

[4]Vascular Biology Section and The Whitaker Cardiovascular Institute, Boston University School of Medicine, Boston, MA

[5]Division of Materials Science & Engineering, Boston University, Boston, MA

[6]Department of Biomedical Engineering, Boston University, Boston, MA

Running Title: Carotid Arteries with Metabolic Syndrome

*** Corresponding author:**

Yanhang (Katherine) Zhang

Department of Mechanical Engineering

Department of Biomedical Engineering

Division of Materials Science & Engineering

Boston University

110 Cummington Mall

Boston, MA 02215

Phone: (617) 358-4406; Fax: (617) 353-5866

Email: yanhang@bu.edu

Table S1. Best-fit values for the 8 parameters of the four-fiber family constitutive model and coefficient of determination, R^2, for each sample. Best-fit parameters are given for both diets (ND and HFHS) for young arteries.

Young – ND

Sample	$c\ (kPa)$	$c_1^1\ (kPa)$	c_2^1	$c_1^2\ (kPa)$	c_2^2	$c_1^{3,4}\ (kPa)$	$c_2^{3,4}\ (kPa)$	$\phi\ (deg)$	R^2
1	20.540	3.742	0.030	11.267	0.043	0.008	1.373	46.352	0.983
2	34.165	1.874	0.068	0.000	1.574	0.025	1.017	52.520	0.982
3	21.143	2.315	0.094	1.327	0.000	0.147	0.744	52.610	0.978
4	28.557	5.030	0.008	13.486	0.000	0.004	2.103	53.172	0.986
5	25.433	5.470	0.022	0.000	0.000	0.728	0.833	56.861	0.983
Mean	25.968	3.686	0.044	5.216	0.323	0.182	1.214	52.303	0.982
SD	5.633	1.593	0.036	6.606	0.699	0.311	0.552	3.778	0.005

Young – HFHS

Sample	$c\ (kPa)$	$c_1^1\ (kPa)$	c_2^1	$c_1^2\ (kPa)$	c_2^2	$c_1^{3,4}\ (kPa)$	$c_2^{3,4}\ (kPa)$	$\phi\ (deg)$	R^2
1	21.855	2.907	0.093	6.387	0.263	0.001	2.403	42.031	0.994
2	36.680	0.320	0.176	0.000	3.772	0.315	0.883	53.986	0.992
3	32.367	0.005	1.371	4.131	0.360	0.002	3.140	42.849	0.989
4	21.761	11.759	0.000	1.159	0.454	0.102	1.398	45.616	0.987
5	27.308	10.403	0.000	4.267	0.000	0.278	0.977	51.128	0.995
Mean	27.994	5.079	0.328	3.189	0.970	0.139	1.760	47.122	0.991
SD	6.549	5.614	0.588	2.577	1.576	0.149	0.979	5.235	0.005

Table S2. Best-fit values for the 8 parameters of the four-fiber family constitutive model and coefficient of determination, R^2, for each sample. Best-fit parameters are given for both diets (ND and HFHS) for adult arteries.

Adult – ND

Sample	$c\ (kPa)$	$c_1^1\ (kPa)$	c_2^1	$c_1^2\ (kPa)$	c_2^2	$c_1^{3,4}\ (kPa)$	$c_2^{3,4}\ (kPa)$	$\phi\ (deg)$	R^2
1	19.770	4.482	0.105	0.005	2.098	0.119	1.303	46.094	0.996
2	19.851	8.629	0.000	0.710	0.726	0.046	1.778	40.755	0.993
3	20.016	1.669	0.176	2.270	0.385	0.267	1.144	48.620	0.991
4	21.484	2.847	0.265	4.262	1.538	0.042	3.657	47.298	0.991
5	13.729	7.785	0.007	6.829	0.154	0.110	1.297	46.153	0.993
Mean	18.970	5.083	0.111	2.815	0.980	0.117	1.836	45.784	0.993
SD	3.012	3.037	0.113	2.776	0.816	0.091	1.046	2.994	0.005

Adult – HFHS

Sample	$c\ (kPa)$	$c_1^1\ (kPa)$	c_2^1	$c_1^2\ (kPa)$	c_2^2	$c_1^{3,4}\ (kPa)$	$c_2^{3,4}\ (kPa)$	$\phi\ (deg)$	R^2
1	22.292	4.466	0.073	0.492	1.427	0.078	2.049	51.032	0.99
2	13.855	4.698	0.019	11.448	0.000	0.036	1.491	49.390	0.994
3	15.089	7.065	0.000	4.220	0.602	0.215	1.172	38.560	0.981
4	17.414	3.695	0.169	2.164	1.052	0.020	3.321	45.373	0.993
Mean	17.162	4.981	0.065	4.581	0.770	0.087	2.008	46.089	0.99
SD	3.724	1.454	0.076	4.825	0.614	0.089	0.947	5.554	0.006

Table S3. Best-fit values for the 8 parameters of the four-fiber family constitutive model and coefficient of determination, R^2, for each sample. Best-fit parameters are given for both diets (ND and HFHS) for old arteries.

Old – ND

Sample	$c\ (kPa)$	$c_1^1\ (kPa)$	c_2^1	$c_1^2\ (kPa)$	c_2^2	$c_1^{3,4}\ (kPa)$	$c_2^{3,4}\ (kPa)$	$\phi\ (deg)$	R^2
1	16.406	18.869	0.000	3.063	0.131	0.004	2.212	40.911	0.982
2	18.602	10.575	0.000	2.171	0.306	0.000	4.687	38.728	0.981
3	6.211	22.814	0.020	18.556	0.000	0.054	4.521	40.112	0.971
4	20.081	17.574	0.000	0.367	0.798	0.082	3.491	41.668	0.992
5	19.578	8.083	0.000	1.400	0.726	0.235	1.898	40.691	0.984
6	23.384	7.071	0.000	0.000	5.184	0.018	3.118	48.084	0.991
7	21.241	11.380	0.532	0.000	0.000	0.019	5.630	44.881	0.984
Mean	17.929	13.766	0.079	3.651	1.021	0.059	3.651	42.154	0.984
SD	5.600	5.992	0.200	6.673	1.864	0.083	1.366	3.226	0.007

Old – HFHS

Sample	$c\ (kPa)$	$c_1^1\ (kPa)$	c_2^1	$c_1^2\ (kPa)$	c_2^2	$c_1^{3,4}\ (kPa)$	$c_2^{3,4}\ (kPa)$	$\phi\ (deg)$	R^2
1	17.023	42.297	0.000	2.987	0.019	0.045	4.957	36.840	0.99
2	21.753	6.302	0.134	1.729	0.785	0.061	2.470	43.103	0.994
3	12.634	25.558	0.000	10.381	0.007	0.958	1.947	40.808	0.976
4	27.585	0.412	0.623	2.595	0.000	0.009	4.312	53.995	0.989
5	26.400	8.210	0.119	0.007	2.799	0.142	2.488	43.237	0.992
6	14.028	6.614	0.019	10.439	0.000	0.031	2.015	40.033	0.986
7	4.554	30.794	0.000	15.334	0.189	1.193	3.530	41.042	0.97
Mean	17.711	17.170	0.128	6.210	0.543	0.349	3.103	42.722	0.985
SD	8.188	15.695	0.226	5.781	1.035	0.503	1.182	5.413	0.009
